# Best practices for an insecticide-treated bed net distribution programme in sub-Saharan eastern Africa

**DOI:** 10.1186/1475-2875-10-157

**Published:** 2011-06-08

**Authors:** Alexis R Sexton

**Affiliations:** 1 Graduate School of Public Health, College of Health and Human Services, San Diego State University, 5500 Campanile Drive, San Diego, CA 92182-4162, USA, Tel 858-603-2946

## Abstract

Insecticide-treated bed nets are the preeminent malaria control means; though there is no consensus as to a best practice for large-scale insecticide-treated bed net distribution. In order to determine the paramount distribution method, this review assessed literature on recent insecticide treated bed net distribution programmes throughout sub-Saharan Eastern Africa. Inclusion criteria were that the study had taken place in sub-Saharan Eastern Africa, targeted malaria prevention and control, and occurred between 1996 and 2007. Forty-two studies were identified and reviewed. The results indicate that distribution frameworks varied greatly; and consequently so did outcomes of insecticide-treated bed net use. Studies revealed consistent inequities between urban and rural populations; which were most effectively alleviated through a free insecticide-treated bed net delivery and distribution framework. However, cost sharing through subsidies was shown to increase programme sustainability, which may lead to more long-term coverage. Thus, distribution should employ a catch up/keep up programme strategy. The catch-up programme rapidly scales up coverage, while the keep-up programme maintains coverage levels. Future directions for malaria should include progress toward distribution of long-lasting insecticide-treated nets.

## Background

Malaria is a re-emerging infectious disease contributing to more deaths in present day than in decades prior [1-4]. Insecticide-treated bed nets (ITNs) remain a highly effective tool in the fight against malaria [1,5-16] when used correctly [4,17-23]. (For the purpose of this paper, the term "ITN" will indicate non-long lasting insecticide-treated nets [LLINs Studies have shown that consistent and correct utilization of ITNs leads to a 90% decrease in the transmission rate [17,24,25].

Use of ITNs hinges on factors of knowledge including the following: causes of malaria, symptoms used to classify malaria, acknowledgement of a causal link between mosquitoes and malaria, traditional mosquito prevention practices, reservations about use of insecticide, and cost of net [26]. Studies show levels of awareness and understanding of ITNs to greatly vary throughout, as well as within, countries. Accordingly, local knowledge and current practices should be accounted for when creating and tailoring an intervention so maximum awareness in any community can be achieved. In general, awareness surrounding malaria is problematic and so community level educational components are necessary to address these deficits.

Studies revealed that despite efforts to create awareness of the causal link between mosquitoes and malaria, significant doubt remained. These doubts were not entirely unfounded as some were based on practical experiences, though others were based on traditional medicine and superstitions [26-28]. Mosquitoes need to be recognized as the sole cause of malaria, prompting a belief that ITNs are a complete defense against transmission and disease. Despite awareness in some areas, low rates of ownership prevailed due to many people believing that ITNs cannot protect against malaria [25]. Given these inconsistencies, it is imperative that any and all misconceptions be rectified through proper education and awareness campaigns. Through this knowledge and awareness, a culture of bed net use, and eventually ITN use, can be created.

In the spring of 2000, the Abuja Declaration was signed by African leaders. This declaration served as a commitment to protect (through provision of an ITN) 60% of African children by the year 2005 [13,17,21,25]. Though this target was not met, universal coverage is still actively sought across Sub-Saharan Eastern African countries [17].

## Review

A search of all available literature on ITN distribution programmes through the Pub Med electronic database (US National Library of Medicine, Bethesda, USA) was conducted using key search terms: bed net, insecticide treated bed net, ITN, ITN distribution programmes, mosquito net, malaria, malaria control, and malaria prevention. Included articlesdiscussed ITN distribution programmes (both large scale and experimental design) that took place in sub-Saharan Eastern Africa from 1996 to 2007. Articles that discussed distribution programmes aimed at diseases other than malaria such as dengue fever or leishmaniasis were excluded on the basis of varying vector behaviour. A total of forty-two articles directly examining ITN distribution programmes were included. Of these forty-two articles, ten were reviews of multiple programmes, including integrated campaigns; five were comparisons of multiple programmes in controlled experimental settings; eleven were single programmes in controlled experimental settings; seventeen were single programmes in large-scale distribution frameworks. Additionally, the search yielded a further sixteen articles of cross-sectional surveys that examined various programmes to provide information on progress, overall impact, and end results. Another sixteen articles were used only as supplemental information on general programmatic components and current distribution trends. Appropriate papers were identified based on abstracts and subsequently reviewed to determine efficiency and effectiveness of ITN distribution frameworks. All distribution frameworks were analyzed and judged on achieved benchmarks such as coverage, cost effectiveness, sustainability, and sound programme components.

## Current trends

### ITN inequities

Current trends show the highest rate of ITN use is generally seen in the target population of children less than five years of age [4,15,16,29,30]. Young adolescents of age five and greater experience a very significant decrease in coverage rate, which is likely due to passing on their ITN to younger children in the family [5,16]. Young adults (ages 20-44) show an increase in ITN coverage likely due to women reaching birthing age and consequently using an ITN for protection of fetuses. Of all age groups, young adolescents ages 5-19 experience the lowest level of ITN protection [30].

Distribution campaigns have led to significant inequities across demographics, especially between rural and urban populaces [11,14,29-34]. While research clearly shows that children less than five years of age have achieved great increases in ITN coverage, many other populations have been left unprotected [11,33,35]. Not only is there a discrepancy in coverage between age groups, there is also a difference between genders within these age groups. Amongst children under five years of age, boys uniformly show greater coverage; directly contrasting adult ITN coverage, which shows that women generally experience greater coverage [15,16,29,31,35]. Coverage generally decreases significantly in the elderly [31]. When there is a level of uniform coverage, greater protection throughout the entire community is achieved [9,30]. Thus, if a programme stands to reach its full potential, universal coverage should be sought.

The majority of the malaria burden falls to the poorest and most rural populations [2,3,13,16,20,29,35-38]. As rural areas are generally more indicative of poverty, significant and alarming decreases in ITN coverage have been observed in these areas [9,21,30,33,35,39]. Matovu and colleagues [29] studied ITN inequities throughout the Tanga district in Tanzania where the predominant sources of ITNs were then commercial retail stores and charitable distribution through the Tanga Rotary Club and Population Services International (PSI) on a much lesser scale (the national distribution campaign had not reached the areas in question at this point in time). It was found that within the children less than five years of age residing in rural locations, 50% were not sleeping under bed net protection while only 14% of their urban counterparts slept unprotected. Of the 50% of rural children sleeping under nets, only 10% of these nets were ITNs compared with 47% of urban children's net being ITNs.

While an uptake in coverage among the less poor populations can be seen in some studies, coverage in the poorest populations generally remains stagnant [27]. During an intervention set in largely rural districts throughout Eastern Zambia, Agha and colleagues [27] found that despite providing the greatest amount of ITN access to the poorest populations, the increase in coverage still occurred in the less and least poor populations. The concept of affordability of ITNs is important. Many distribution programmes aim to increase access to ITNs without adequately addressing cost. This approach will not yield an increase in overall coverage because the issue of cost is still present.

To achieve equity between socioeconomic statuses, distribution programmes need to concentrate on targeting rural locations as opposed to easier to access urban areas [35]. This shift in focus may lead to logistical issues including how best to reach these remote populations, how to tailor cost of ownership to the financial means of the population, among other reasons. Free distribution programmes access the poorest populations with the greatest success. However, free distribution also requires a fair amount of financial backing. That being said, in order to produce a sustainable distribution programme, it is necessary to walk a very fine line between large subsidy and small cost recovery schemes.

### Barriers to ITN use

Research overwhelmingly shows that the most prominent barrier to ITN ownership is cost [1,11,15,27,29,36,37,40-42]. The average annual income in sub-Saharan Africa is estimated to be $1 per day per person [1,20]. Consequently, especially in the poorest populations, people are far more likely to allocate financial resources to items deemed immediate and essential (i.e. food, clothing, kerosene) [28,29,36].

Matovu and colleagues [29] assert that knowledge (or lack thereof) and overall beliefs about ITNs are a considerable barrier to ITN use. During a focus group, one participant stated: "*When you look at us and the clothes we are wearing, would you really think we cannot buy ngao [insecticide] or a bed net? For me I think we don't have good knowledge about those things and how important they are in fighting malaria*". This sentiment was echoed by the finding that oftentimes cost is not the only barrier discouraging ownership; lack of value attached to ITNs plays a large role. Without proper knowledge, populations are unable to connect malaria prevention and ITNs [22,25,28]. Thus, it is necessary not only to clarify that ITNs solely prevent malaria, but also to illuminate the direct benefits of ITN use in terms of the financial costs averted through less hospital visits

Distance and accessibility to ITN distribution points also serve as barriers to ownership as access to distribution posts still remains scarce in many countries and communities [5]. The likelihood of ITN purchase is inversely related to the distance from an ITN distribution point [1,15,24,43]. This inverse relationship is largely responsible for the drastically lower rates of ownership in rural communities. Without reasonable access, let alone equitable access, populations removed from more urban/commercial centers will largely never encounter ITN ownership opportunities.

### Stakeholders

Stakeholders derive from a variety of organizations, foundations, companies, researchers, etc. However, these various groups differentiate further into two distinct categories: public and private. Typically many programmes amass a mixture of these stakeholders to sustain adequate backing. Through bringing together multiple and diverse stakeholders, programmatic issues stand to be addressed by experts within the given specialty, thus leading to a stronger and more efficient campaign [23,40,44].

An exemplary mixed stakeholder programme was behind the Tanzania National Voucher Scheme (TNVS). The TNVS programme encompassed at least ten distinct groups of stakeholders from both the public and private side. To name a few: Ministry of Health National Malaria Control Programme, World Vision/CARE, health facilities, Swiss Tropical Institute. Each stakeholder took responsibility for one of the varying roles such as: activities funder, project manager, social marketing and technical support, handling vouchers, top-up charges for ITNs [45]. Multiple stakeholder schemes like the TNVS are not unusual as many programmes spread responsibilities over a host of different organizations. This serves to ensure programme stability and expertise [45] as these stakeholders collaboratively determine everything from the amount of resources available to the logistics of the actual programme.

### Private sector

The private sector stands to offer greater resources in terms of financial backing, labour support, technological advances, and more. This type of support simply cannot be offered by domestic governments as resources are already scarce. The private sector can contribute everything from financial resources to market research, and even product development [23,40]. However, interests and expectations between the private sector and public health policymakers often differ [46], sometimes causing friction that stands to halt or severely cripple progress. To circumvent this ideological gridlock common goals must be agreed upon prior to the programme launch.

Commonly, resources provided by the private sector are the tangible health products; in the instance of ITN distribution, ITNs. A distribution programme is then structured in accordance with stakeholder agreement. Not surprisingly, the private sector oftentimes centralizes distribution in urban areas where buying power is the least limited (contingent upon a subsidized top-up programme), despite the fact that the more rural and poorest populations are best served by this resource [46]. In this way, private sector run distribution programmes do not prove the most beneficial and effective for rural and poor populations.

### Public and commercial sectors

Public stakeholders deal largely with the consumer, or in this case, the ITN user. These stakeholders aim to provide the greatest amount of assurance and protection to the consumer as possible. Quite importantly, the public stakeholder creates and stimulates commercial demand [40]. An increased demand forces the commercial sector to maintain competitive prices, enabling a greater population to afford ITNs.

It is evident that the public and commercial sectors are significantly intertwined as neither can elicit high ITN coverage rates without the other [40]. Accordingly, these sectors must work together to bolster ITN demand, supply, and availability. Factors that will ultimately enable this kind of scenario include eradication of taxation on ITNs, quality control between ITN producers and suppliers, and equity in accessibility of ITNs [40].

### Sustainability

Many distribution programmes initially increase ITN coverage; though a hallmark of an exceptional programme is sustainability in distribution and coverage. Utilizing existing resources is an effective method to achieve sustainability, though currently, these existing resources are greatly underutilized [45]. When existing resource opportunities are exploited, distribution costs generally decrease greatly, thus increasing overall programme sustainability [42].

Programmes that heavily rely on sole donors greatly hinder any possible sustainability. This is due to the changing financial climate which does not allow for long-term financial commitments [45,47]. An example of the need for programme sustainability and reduced reliance on donors occurred in Kenya during late 2005 to early 2006. Free ITN distribution took place through twenty-eight ANCs, distributing 17,893 ITNs across the country. However, distribution came to a prolonged halt when one donor revoked commitment. Distribution was not resumed until another donor and subsequent funding was secured [48]. Programme stability aims to avoid and eradicate situations such as this. Instead, a mixed approach should be employed when possible to ensure longevity and effectiveness [47].

In 2002, Brentlinger and colleagues [49] attempted a distribution programme in Sofala and Manica, two central provinces of Mozambique. The programme was a collaborative effort with the domestic Ministry of Health (MOH). Researchers envisioned the programme as a free distribution design despite the MOH preferring a for sale design to ensure cost recovery. That being said, researchers were forced to find additional funding. Given the short notice, the secured funding was not surprisingly less than stable. This resulted in a several month long blackout of freely distributed ITNs when funding was suddenly cut. Ultimately, funding was again procured, though not in an amount which allowed any cost recovery as the researchers and MOH initially planned.

### Distribution cost frameworks

As there is no consensus to the preeminent ITN distribution framework, researchers and programme directors utilize an array of approaches with common goals. Each approach can be categorized largely upon the fundamental cost recovery scheme, generally ranging from free distribution (no cost recovery) to varying degrees of subsidized distribution [34,36,42,46]. It should be noted that cost recovery does not necessarily ensure programme sustainability or denote low equitability [42].

### Subsidized distribution

Through lowering ITN cost below market value, more of the target population gains purchasing ability. Subsidies enable these below market prices and are generally provided by governments or external donors (i.e. private organizations, NGOs) [13]. For a majority of populations, subsidies are the only way purchasing ITNs becomes feasible [20].

Partially subsidized distribution is currently the most popular framework throughout Africa [12], proving successful in a number of settings [10,13,32,38,47]. Subsidized distribution is a cost sharing mechanism through which donors pay a portion of the cost leaving the remainder of the cost to the buyer. It is a strategic move to lower the cost of ITNs, thereby enhancing purchasing power within the population and bolstering community wide protection [32]. While partial subsidization is not as equitable as free distribution, it does promote higher equitability among poorer populations [13,14]. Moreover, it often proves much more sustainable than a free distribution scheme as a portion of the distribution cost is regained [13,14].

The amount or level of a subsidy is generally determined by several key factors including size of the target population, ability and willingness to pay, as well as resources available for subsidization [23]. While subsidies provide general support to the poorer populations and so increase equity, there is concern that they may negatively impact the commercial ITN sector [38]. This is cause for some alarm as the commercial sector shows the most potential for long-term sustainability and overall bolstering of the economy [38].

### Free distribution

As cost is the major barrier to ownership, free distribution should be considered for the poorest of poor [11,16]. Not only is free distribution the most equitable approach to ITN coverage, it also stands to provide more immediate results as the population is not required to do anything more than attend the distribution post and receive an ITN [22,33,47,50]. Some proponents rationalize that ITNs are public goods and so should be provided at no cost to all, or at least to those most susceptible to infection [16,23,37,42,45,51,52].

Noor and colleagues [34] observed the greatest increase in ITN coverage among populations which enjoyed free distribution. Further research shows that free distribution covers the highest proportion of the population designating it as the most equitable framework [14,29].

Some research favors the idea that free distribution should only be applied to populations experiencing the most devastating poverty [14,16]. Though this type of selective dissemination is logical in theory, it is complicated to define and identify the poorest populations. Operationally, it proves easier to extend free distribution to easily identifiable groups, i.e. the most vulnerable populations to malaria fatality: pregnant woman and children five years of age and younger [11]. Mathanga and colleagues [12] embarked upon a pilot study in Malawi which integrated immunization services with free ITN distribution. Pursuant to completion of the pilot study, health workers reported a noticeable decrease in malaria cases. Research findings supported these observations as results showed a nearly fourfold increase in one to two year old children sleeping under ITNs. As a direct result of this pilot, Malawi enacted malaria control guidelines dictating that all ANCs distribute ITNs to pregnant women and children less than five years of age.

While many favor free distribution for the various benefits already discussed, there are indeed drawbacks. Free distribution requires a great deal of donor support and resources. It is a logical concern that free distribution cannot prove sustainable due to the extent of its high resource demand [14,18,29,42,45]. Another concern is that while it clearly serves the poorest populations more effectively than any other framework, it also benefits the populations who are more financially well off as they continue to receive assistance through subsidies [29]. As a result, an increased strain on already limited resources ensues, exacerbating the over reliance on donor support.

### Commercial distribution

Commercial distribution is entirely user financed. This means that the buyer is responsible for the full cost of the ITN as well as the added cost of distribution to the supplier [46]. Though this seemingly defeats the purpose of a health promotion programme, it remains important to discuss the role of the commercial sector in ITN production. Ideally, the least poor populations should purchase ITNs through the commercial sector thereby stimulating growth and in country production [16,46,53].

Though commercial distribution does not alleviate all cost barriers to the population, the framework is sustainable [13,40,47,54] as it does not rely on any outside funding [16]. Researchers have found that commercial retailers are actually responsible for the vast majority of nets amongst the general population. Between one half and two-thirds of the population obtain nets through the commercial sector with no subsidy (it should be noted that the least poor purchase the unsubsidized ITNs more frequently than the poorer populations) [15,25,30], showing that it may be a sustainable framework and an important aspect of ITN distribution and coverage [13,47].

### Distribution pathway

The WHO advocates a distribution approach where emphasis is placed on the commercial sector. Accordingly, the highest subsidies should be provided to those in need of the greatest financial support. However, this framework fails to account for the true extent of poverty. Under this scheme, heavy subsidies would theoretically be almost universal as poverty rates across Africa are continually rising [41]. Resources needed to launch and sustain a programme such as the one WHO advocates are extensive and not easily secured [41].

### Antenatal clinic

Though it seems intuitive that households with pregnant women would be more likely to own ITNs, it has been found that presence of a pregnant woman is not necessarily indicative or associated with ITN ownership [49]. As pregnant women are in the target population, it is necessary to create avenues through which this specific population can achieve adequate coverage.

As African women generally share a bed with their infants and small children, ANCs provide existing infrastructure to disseminate ITNs to all individuals within the target population [23,45,48,55]. Furthermore, a majority of women (greater than 70%) in sub-Saharan Africa utilize ANCs at some point during pregnancy [10,33,48,51,55,56], and so the majority of pregnant women would come into contact with at least one ITN programme if all ANCs harboured distribution programmes. With the increase of ANC based distribution programmes, researchers are also finding that pregnant women are visiting the clinic earlier during their pregnancy (the average sub-Saharan African woman visits a clinic at week twenty of pregnancy [55]) in order to obtain an ITN or ITN voucher [4,23,40,50]. This result is two-fold: women are receiving care and guidance earlier on in pregnancy while also protecting their unborn children from malaria transmission over longer and possibly more vulnerable stages during pregnancy.

ANCs provide existing infrastructure to reach the target population and disseminate ITNs. By exploiting this existing resource, programmes can contain or significantly reduce costs [45,48]. In this way, resources which can be distributed into other aspects of a programme for greater coverage achievement are freed up, leading to greater potential for programme sustainability. When distribution through ANCs is coupled with free distribution, the outcome is a simple yet effective and equitable distribution framework [11,36,48].

As with many distribution programmes, equitability between the urban and rural, or relatively wealthy and poorest populations proves challenging. Rural populations are not as likely to utilize ANCs as they are faced with issues relating to accessibility of the clinic. Furthermore, any ANC ITN distribution programme (without in-clinic ITN distribution) would be met with the same issues of accessibility to the ITN distribution center as well as heightened financial constraints [33].

Distribution through ANCs is not without complications or flaws. Oftentimes, women report that they were not provided with accurate information surrounding ITN use. An example was observed by Guyatt and Ochola [57], when a woman reported that the ANC staff instructed her to save the ITN for use after she gave birth; this led her to abstain from ITN protection during pregnancy. Not only is inaccurate and misleading information sometimes provided, but this process puts the ANC staff in control of providing the ITNs and so hinges upon the staffs' own biases of eligibility [6,23].

Proper training of health workers proves to reduce the overall incidence of malaria [58-60] as correct information and techniques are being provided to the population. Without proper instruction and training, many health facility workers do not entirely understand their roles in distributing ITNs. This causes incongruities as differences in distribution arise between clinics, creating an environment where equity is not really possible [42].

Several reservations about distribution through ANCs exist. In smaller, more rural ANCs there are less staff members. Many of these areas have a nurse-to-patient ratio of one nurse to a few thousand patients [25]. It may, therefore, prove difficult for such a small work force to carry out the actual duties of their position in addition to added tasks from the programme [23,49,50]. This proves to be a legitimate concern as programme representatives cannot reasonably be stationed at all ANCs. Then again, if the task solely consisted of providing patrons with a voucher as opposed to an educational session, this may prove less of an issue. Still, it is ideal that staff take the time to educate pregnant women regarding the importance of protecting themselves and their unborn children through use of an ITN regardless of whether it is programme mandated or not. Issues of physical space for ITNs are a concern as well [49]. ITNs are bulky and require adequate storage space if physically distributed through an ANC. Many health facilities are already severely limited in space and so cannot spare any space for the storage of ITNs.

### Catch up or integrated vaccination campaign

Throughout Africa various childhood immunization programmes are considered the gold standard for health services as they are particularly efficient and effective. Moreover, they are capable of reaching and protecting the vast majority of youth throughout the region; 95% in some areas [6,19,29,37,59,61]. The established infrastructure of these programmes provides a useful venue through which to add additional health services such as ITN distribution. The existing infrastructure enables planners to easily orchestrate programmatic details such as location(s), time, and dissemination [11,59]. With less necessary energy exerted on infrastructure development, other programme details may be more thoroughly developed or emphasized. To date, there have been no reported adverse effects to immunization campaigns due to an added ITN distribution [61]. As there are no obvious or known negative repercussions stemming from campaign integration, more programmes of this nature should be employed.

These integrated campaigns (often referred to as catch up campaigns) are not only proven to be just as effective as voucher schemes, but also more cost effective [19]. This results from the utilization of a direct distribution framework which eliminates the high cost of transportation and exchange of vouchers. Transportation and voucher exchange require resources including: vehicles, gas, drivers, and travel time. These expenditures can be invested in other programme aspects such as greater ITN quantities when transportation is eradicated.

Subsequent to an integrated ITN distribution/immunization campaign in Malawi, Mathanga and colleagues [12] found a two to three fold increase in the amount of children (ages 12-23 months) who slept under an ITN and were fully immunized. When this increase was compared to the control area (no change in the proportion of children both fully immunized and sleeping under an ITN), there was a clear and positive impact resultant of the integrated campaigns. Higher coverage in both health service intervention goals (childhood immunization and ITN distribution) was evident.

Skarbinski and colleagues [59] implemented a free distribution of ITNs through a measles vaccination campaign in the rural region of Lindi, Tanzania. Concurrent research showed that 96% of the households surveyed were previously aware of the integrated campaign. Awareness was generated through community leaders, neighbours and radio announcements. When polled for motives for campaign attendance, over 60% of participants attributed attendance to the free ITN. This particular campaign resulted in a 16% rate of ITN ownership increase (from 53% to 69%), and a significant equity increase between the poorest and wealthiest quintiles. This study highlights the integrated campaign approach as a useful means to promote equitability, especially in terms of rapidly scaling up coverage.

Though distribution through vaccination programmes garners high coverage, it should be noted that these programmes are insufficient in maintaining coverage. Vaccination programmes are conducted rather infrequently, sometimes taking place only every few years. Consequently, children born after the programme date as well as pregnant women [19] will remain unprotected and vulnerable to malaria transmission until the next immunization programme (assuming an ITN is not already in the home). A supplemental programme is needed as integrated campaigns do not guarantee a sustained high coverage level [6,12,19].

### Physical distribution logistics

Physical movement and transport of ITNs is an important logistical factor to account for during programme planning. Many unallocated resources can quickly be depleted if proper transport logistics are not addressed. Travel within Africa is generally inefficient due to the lack of quality roads and accessible sea ports. Transportation within the target community is not without logistical issues either. ITNs prove large and bulky as cargo. Due to the poor quality in roads, versatile but compact four-wheel-drive trucks are relied on which reduces the amount of ITNs that can be transported at once. Having the foresight to see the logistical complications of ITN transportation throughout Africa will allow for more accurate budgeting and efficient trip planning [49]. Courier programmes willing to provide services to distribution campaigns have readily agreed to deliver ITNs in urban areas, though only agree to deliver to the easily accessible rural areas [15]. This disparity again highlights the struggle for equitability between the urban and rural populations.

### Categorization of delivery systems

Programmes ranging from public sector free ITN distribution (Malawi), to integrated campaign free distribution (Togo), to private sector partially subsidized distribution (Senegal), to mixed public/private partially subsidized distribution through a voucher scheme (Tanzania) have been implemented with a varying range of success [50]. Though all programmes merit some praise, it remains difficult to determine which design is most effective and adaptable.

### Voucher delivery

Voucher systems rapidly increase community ITN coverage [10,29,40]. The structure behind the voucher scheme is rather simple though generally employs a more complicated mixed public-private structure approach [10]. First, the voucher (subsidy) is handed out to the target population through a distribution channel (public ANC, school, etc.). The recipient then presents the voucher to a participating ITN retailer (private or commercial sector) and is able to purchase the ITN at a subsidized price [4,23,30,38,40,47]. This design is advantageous for a number of reasons. It alleviates health facilities of the burden of physically handling ITNs and payment. It also provides flexibility and greater choice in ITN purchase, all the while strengthening the commercial ITN market [23,40,60].

The TNVS is arguably the most well established voucher distribution scheme. It was launched in late 2004 by the Tanzanian Government in conjunction with the Global Fund to Fight AIDS Tuberculosis and Malaria [4,47]. The TNVS was among the first voucher systems and remains one of the only programmes that is country wide.

A seemingly major flaw in most voucher systems is logistical in nature. The voucher is received at one place (likely an ANC) and the ITN is received at another (local retailer). Some participants find the need for separate trips inconvenient or impossible if they live in rural areas [4]. This suggests a need for change; for example, all ITNs could be sold at the ANCs or other voucher distribution areas [38] eliminating the need for a second trip. In this exact way, the TNVS has managed to skirt the inconvenience. The TNVS ITNs move only from the net producer, wholesaler, or retailer to the ANC, removing much of the transport needed in other programmes. More importantly, participants are able to obtain their voucher and ITN in one location eliminating the inconvenience of multiple trips and wasted time.

The ability of a voucher system to track ITNs is very effective for overall programme evaluation. For example, Mushi and colleagues [13] found that after two years of programme implementation in Tanzania, a very small portion of the target population never received a voucher. This fact, despite data showing 100% of people who received a voucher subsequently redeemed their ITN, reflects a chasm between certain study indicators. As a result, it was inferred that the rate of ITN leakage was incredibly high, meaning people who were not intended to receive a voucher in actuality did. Without the monitoring ability of a voucher scheme, this rate of leakage could not be so easily detected. Programmes can reassess voucher distribution practices to ensure ITNs reach the target population in the next implementation. Other studies find this same high rate of voucher return [4,10] demonstrating stronger accountability at voucher distribution centers is needed.

Ostensibly, a voucher system seems fairly straight-forward. However, complications arise and so there are undoubtedly corresponding issues. As previously mentioned, vouchers do not consistently reach the entire target population. This may be due to staff biases, lack of staff training, understaffing, among other reasons [10,38]. Tami and colleagues [38] interviewed staff at a voucher distributing clinic and found that a common practice was to provide vouchers only to women who expressed an intention to purchase an ITN. Additionally, if a woman failed to produce her ANC health card, she was declined the voucher. In the same study, the staff stated that they felt they needed more information on malaria, ITNs, and how the voucher system worked. Moreover, they felt that ITNs should be sold within the clinics at the discounted rate. Justifications of withholding vouchers from other studies include the belief that the woman was not able to pay the top up fee for purchase of the ITN [4,10] and the woman already owned a net and thus did not need another [10]. These statements reflect a need for clear levels of communication between voucher center staff and ITN stakeholders.

Through educating women of the target population about the ITN programme and malaria, it may become more difficult for ANC staff to selectively withhold vouchers [23,38]. This prompts the need for an educational component which stands to clarify eligibility criterion and the population the programme intends to serve [13]. With an increased emphasis on promotion and awareness, the uptake of the voucher programme may quicken. Measures including point of sales advertisements, mass media campaigns, and health clinic promotion campaigns all stand to garner greater awareness of the voucher programme. Increased awareness and overall programme comprehension is important as studies show that uptake of participation in voucher programmes is relatively slow at three to four years on average [13,33]. Within ANCs and other distribution centers it is important that supervision of voucher distribution is strong [4,38]. Proper supervision leads to less misuse of vouchers and further promotion of the programme, enabling the target population to purchase more ITNs. Tami and colleagues [38] suggest that intermittent audits of distributed vouchers be conducted to ensure correct use. These audits may include tracing the voucher to the recipient in order to confirm ITN redemption.

### Long lasting insecticide-treated nets and retreatment

Long-lasting insecticide-treated nets (LLINs) are becoming widely available. Distribution programmes should transition ITN supplies to LLINs in order to maintain high coverage levels for longer durations. This eradicates the need for treatment/retreatment programmes as the insecticide in LLINs lasts throughout the life of the net [33,44,49,59]. The resource expenditures used to continue social marketing treatment/retreatment programmes could then be redirected to other aspects of the programme thereby creating strength in other areas. While this is an ideal strategy, it will likely not be feasible for some time in the poorest and rural areas.

In the interim, treatment and retreatment programmes remain necessary in order to maintain coverage levels of regular ITNs. A compelling programme structure was introduced by Guyatt and colleagues [62] in ANCs across Kenya. Though this programme was not specifically marketed as a retreatment programme, the basic structure can easily translate to such. ANCs provided nets along with sachets of insecticide for treatment at home. A retreatment programme can be designed accordingly with the sachet of insecticide as well as instructions intended for retreatment six months subsequent to receiving the ITN. The main drawback to this proposal is there is a likely chance that the sachet could be lost, forgotten, or misused. In the above mentioned Guyatt *et al *study, it was found that a significant amount of recipients (13%) were never provided with treatment instructions. Though, it is an inexpensive design that can work until LLINs are made widely available.

### Universal coverage

While it is undisputed that pregnant women and children younger than five years of age are the target population for malaria prevention, there is a shift toward universal coverage. Spatial analysis supports the concept that community wide protection through ITN coverage will only be reached when a significantly high portion of the community is indeed covered. ITN and LLIN distribution, therefore, should target the entire population in order to reach community wide benefits [4,30,32,61,63].

The target population only accounts for 20% of human to mosquito interplay, leaving the remaining 80% to occur in the majority of the population [9]. Within this non-target population is almost the entire work and labour force of a given country. The labour force is responsible for the economic productivity and growth of the country. Thus, it stands to reason that the labour force should be protected in the interest of preserving the economy [4,15,30,51]. Others at-risk for heightened transmission are the immune compromised populations (HIV/AIDS, tuberculosis, malnourished). They should also be covered as they are particularly susceptible to acquiring fatal malaria [30,48].

### Monitoring and evaluation: programme evaluation and effectiveness

Programme evaluation is a vital part of any programme and should be implemented as early as the planning phases. However, monitoring and evaluation can serve a different, though equally important function for ITN distribution programmes. Through consistent monitoring and evaluation, researchers are able to determine potential sustainability for many programmematic aspects including distribution channels, resources, and supplemental campaigns [10,23,47]. While monitoring distribution and movement of ITNs throughout African countries is not always an easy process [42] it is nevertheless crucial to understanding the efficacy of a distribution programme.

Voucher programmes provide an excellent means of monitoring the number of ITNs that actually reach the target population [10]. This framework proves the most conducive to a monitoring component. The distribution pathway (ANC) is able to record the number of vouchers handed out. The programme researchers can then count and record how many ITNs were actually redeemed as the voucher is exchanged to the retailer for the ITN. The retailer then provides all claimed vouchers back to the programme [38] for an overall count.

A well developed monitoring and evaluation system was implemented by the TNVS. It includes five domains of monitoring: ITN coverage among target population, use on ANC services, leakage of vouchers and ITNs, commercial ITN distributors, and cost effectiveness [60]. Leakage occurs when an ITN is redeemed through the programme by any person outside of the target population. It should be noted that ITN leakage is not the same as wastage. These ITNs are ultimately being used for the prevention of malaria transmission and as such are still lowering the overall malaria incidence and prevalence rate [42]. These domains serve to provide the following features within the monitoring and evaluation programme: independence, breadth, attention to time, coverage of all processes, attention to leakage, and acute notice of changes [60]. This monitoring system is simple and should serve as an example for other programmes aiming to create a monitoring system.

### Next steps

#### Unsuccessful programmes and components

Although no common ground has been reached in terms of a best practice for distribution, it is important to internalize previous failures within the field and improve from there. When attempting to create a sustainable programme, cost recovery and sharing are necessary, accordingly so is a strategy to promote equitability. Many have relied on social marketing as a practice to induce this; however it does not appear successful. Agha and colleagues [27] showed social marketing as unable to effectively reach the most rural and poor of communities as there was not enough of a subsidy to promote purchasing power. Studies have shown that inequalities in net ownership are not addressed or alleviated in a pure social marketing framework [29], nor is the increase in ownership of ITNs as rapid as in other campaign frameworks. Social marketing can prove more costly per ITN distributed than commercial sector distribution (without programme assistance) though may lead to greater coverage of the target population if employed correctly. Given that the cost of a socially marketed ITN can be two times higher than a commercial ITN [46]; the benefits of social marketing must greatly outweigh the increased cost.

## Conclusion

Though ITN distributions aimed at a rapid scaling up are imperative to increasing coverage, these efforts must be used in conjunction with regular and routine access to ITNs for the entire population [14]. This will ensure the highest level of coverage, ultimately yielding the highest decrease in malaria burden. It is necessary to note that different and simultaneous delivery tactics/programmes do not counteract one another, but rather complement each other through creating greater levels of overall coverage [6,30]. This synergistic effect leads to a more rapid scale up of coverage and should thus be adopted. The most logical multiple approach is a catch up/keep up strategy. The inclusion of ITN distribution during routine immunization day campaigns (catch-up) coupled with continued ITN distribution through ANCs (keep up) [33] ensures the greatest coverage of the target population (though does not address universal coverage). Within this strategy, rapid scaling up of coverage occurs through the catch up portion while distribution through the keep up portion sustains the achieved high coverage [4,6,33,50].

Distribution programmes that reduce both cost of ITN ownership and distance to point of ITN distribution points see greater increases in coverage [43]. Though a greater amount of people are able to afford and access ITNs in such programmes, it does create problems of feasibility due to programme resources as greater amounts of funding are necessary to decrease cost of ITNs and increase the frequency of distribution points. However, through using existing resources such as infrastructure already in place (for example, ANCs or long-standing immunization campaigns); programme costs can be kept minimal thus enhancing sustainability [42]. Programmes need to assess all existing resources in order to minimize unnecessary expenditures so that ITN costs can be kept to a minimum and more distribution posts can be provided.

Ultimately, the need for a strong educational component to any distribution programme is necessary to explain and promote the benefits of ITN use. This education should also include proper use, hanging, and care of ITNs [52]. Awareness and knowledge of benefits and proper use of ITNs will lead to the most consistent and correct use, consequently increasing coverage and community wide benefits. The life span of each ITN may also increase through proper care thereby lowering overall lifetime costs of ITNs per person.

### Best practice

The considerable variation of ITN distribution programmes provides a wealth of frameworks to draw from. However, this same lack of uniformity serves to differentiate programmes to the point that exact programme replications are few and far between. That being said, a theoretical programme based on integral components within the most successful programmes can be deduced from the literature and research.

The studies concur that a free distribution catch up programme (in areas greatly in need of rapid coverage increase) in conjunction with a universal coverage, modest subsidy/cost-sharing programme as a means of keep up is the most efficient and effective means of controlling malaria transmission in sub-Saharan Eastern Africa. The modest level of cost sharing ensures greater programme sustainability while still lowering cost of ownership and increasing purchasing power within the populace. Once ITN coverage reaches the community wide benefit threshold, non users will experience protection at a near equivalent level as actual ITN users. This is compelling reasoning for a universal coverage programme. Formative research into the programme population will provide insight as to how best a general programme framework can be tailored to any specific population or generalized to any larger populace.

Looking ahead, all ITNs should be phased out in favor of LLINs when economically feasible. Furthermore, ITNs should be procured from domestic producers when possible. Domestic procurement invigorates both the commercial market and the economy thereby generating even greater sustainability. This economic stimulation will ultimately lead to a positive trend within the country serving to better the overall quality of life. The ITN distribution programme will not only prevent and control malaria transmission, but also enhance the overall condition of the country and subsequently, the quality of life.
